# Antiviral Activities of Interleukin-27: A Partner for Interferons?

**DOI:** 10.3389/fimmu.2022.902853

**Published:** 2022-05-10

**Authors:** Heather Amsden, Olena Kourko, Madison Roth, Katrina Gee

**Affiliations:** Department of Biomedical and Molecular Sciences, Queen’s University, Kingston, ON, Canada

**Keywords:** interleukin-27, interferons, virus, viral immunology, infection, antiviral immunity

## Abstract

Emergence of new, pandemic-level viral threats has brought to the forefront the importance of viral immunology and continued improvement of antiviral therapies. Interleukin-27 (IL-27) is a pleiotropic cytokine that regulates both innate and adaptive immune responses. Accumulating evidence has revealed potent antiviral activities of IL-27 against numerous viruses, including HIV, influenza, HBV and more. IL-27 contributes to the immune response against viruses indirectly by increasing production of interferons (IFNs) which have various antiviral effects. Additionally, IL-27 can directly interfere with viral infection both by acting similarly to an IFN itself and by modulating the differentiation and function of various immune cells. This review discusses the IFN-dependent and IFN-independent antiviral mechanisms of IL-27 and highlights the potential of IL-27 as a therapeutic cytokine for viral infection.

## Introduction

The emergence of novel viral threats, such as the current COVID-19 pandemic, has highlighted the importance of viral immunology. Understanding of the immune response during viral infection sheds light on aspects that can be manipulated with vaccines and therapies to enhance antiviral activities. Within the immune defenses, Toll-like receptors (TLRs) are among the four major sub-families of pattern recognition receptors (PRRs) capable of recognizing pathogen-associated molecular patterns (PAMPs) (1). Viral PAMPs activate TLRs expressed by antigen presenting cells (APCs) to produce soluble mediators, such as cytokines (2). Cytokines influence how antiviral responses are initiated by innate immune cells and maintained by adaptive immune cells, orchestrating immune responses that lead to favourable or detrimental outcomes. For instance, sufficient cytokine-induced inflammation and immune cell recruitment is crucial for viral clearance, however, overproduction of cytokines can lead to excessive inflammation and tissue damage, characteristic of a cytokine storm (3). Therefore, understanding the complex regulations and actions of cytokines is crucial in further understanding antiviral responses and developing anti-viral therapies.

Produced in response to TLR activation, interleukin-27 (IL-27) is a cytokine of interest for its activity against viral infection. IL-27 is composed of two subunits, IL-27p28 and Epstein-Barr virus-induced gene 3 (EBI3) (4), and signals *via* a heterodimeric receptor consisting of WSX-1 and glycoprotein (gp130) (5). IL-27 belongs to both the IL-6 and IL-12 superfamilies of cytokines as it shares the gp130 subunit (IL-6 family) and is heterodimeric in nature (IL-12 family) (6). Binding to its receptor predominantly activates Janus kinase 1 and 2 (JAK1 and JAK 2), which then phosphorylates signal transducer and activator of transcription (STAT) 1 and 3 (5). Tyrosine-phosphorylated STAT1 and STAT3 dimerize and translocate to the nucleus to activate transcription of various genes. Due to the similarities in structure of cytokine and receptor subunits of IL-27 with IL-6 and IL- 12, this cytokine was expected to be pro-inflammatory, which was emphasized by early studies demonstrating the ability of IL-27 to promote NK and T cell proliferation and production of IFNγ (4, 7). Later studies highlighted additional mechanisms governed by this cytokine, including, inhibiting Th2 and Th17 cell activities, and anti-inflammatory functions such as stimulating the production of IL-10 by T cells (8, 9). Within innate immunity, IL-27 can upregulate TLR expression and function in myeloid cells (10–15).

The vast immunomodulatory properties of IL-27 link innate and adaptive immune responses, and have made it a cytokine of interest for developing novel antiviral therapies and adjuvants for vaccines (16, 17). Evidence of the potency of IL-27 as an antiviral cytokine has been accumulating over the past decade and demonstrates that IL-27 can inhibit a wide range of viral infections including human immunodeficiency virus (HIV), hepatitis B virus (HBV), hepatitis C virus (HCV), herpes simplex virus (HSV), influenza, zika virus (ZIKV) *in vitro* and *in vivo (* 18–30) (**Table 1**). Interestingly, the antiviral functions of IL-27 also parallel those of IFNs, and with evidence that IL-27 and IFN each possess the ability to induce expression of the other (18, 20, 28, 29, 36, 39, 40, 43, 44) it is important to consider how these cytokines may act synchronously or asynchronously with one another. In this review, we discuss the antiviral effects of IL-27 by broadly grouping these effects into IFN-dependent and IFN-independent mechanisms.

**Table 1:** Evidence of IL-27 inhibition of viral infection.

Virus	Model	Mode of inhibition by IL-27	Reference
ZIKV	Primary human keratinocytes	Activation of STAT1 leads to *OAS2* transcription independent of type I and type II IFNs	Kwock et al. (19)
HBV	Human hepatocyte cell lines (HepG2, Huh7)	Increases type I and III IFN production leading to *OAS1, PKR*, and *MX1* transcription	Cao et al. (20)
		Complexes with IL-6R to inhibit infection	Yang et al. (31)
	Human kidney cell line (HEK 293), human hepatocyte cell line (HepG2)	Type I IFN-mediated IL-27 production induces TRIM25 expression	Tan et al. (21)
HIV	Primary human MDMs	Increases IFNα production leading to enhanced APOBEC cytidine deaminase expression	Greenwell-Wild et al. (23)
		Reduces SPTBN1 expression independently of IFNα	Dai et al. (24)
		Increases transcription of ISGs such as *MX1, OAS2*, and *PKR* similar to that of IFNα	Imamichi et al. (32)
	Primary human monocyte-derived DCs	Increases transcription of ISGs such as *MX1* and *OAS2* independently of type I IFNs	Chen et al. (25)
HCV	Human hepatocyte cell line (Huh7.5)	Inhibition partially dependent on IFNα	Frank et al. (26)
	Mice	Increases HCV-specific IFNγ-producing CD8+ T cells synergistically with IL-12	Matsui et al. (17)
IAV	Human hepatocyte cell line (HepG2)	Increases transcription of *MX1* independently of IFNα and IFNγ	Bender et al. (27)
	Primary human PBMCs and lung epithelial cell line (A549)	Induces IFNα production which leads to expression and activation of PKR	Liu et al. (29)
	Human lung epithelial cell line (A549)	Complexes with IL-6R to induce type I and III IFNs which leads to increased ISG transcription	Zuo et al. (33) Yang et al. (31) Wang et al. (34)
	Mice	Augments NK cell cytokine production and effector functions	Kumar et al. (28)
		Increases IAV-specific IFNγ-producing CD8+ T cells	Mayer et al. (35)
		Mediates immunopathology by promoting T-cell production of IL-10	Liu et al. (18) Jiang et al. (36) Sun et al. (37)
CHIKV	Primary human MDMs	Inhibits infection in the absence of IFNs	Valdés-López et al. (38)
LCMV	Mice	Promotes pDC differentiation and NK cell effector functions	Harker et al. (39)
HSV-1	Primary human macrophages and DCs, human epithelial and glioma cell lines (HeLa, U373MG, and T98G)	Enhances proinflammatory cytokine IL-6, IP-10 and MIG production	Heikkilä et al. (40)
	African green monkey kidney epithelial cell line (Vero cells)	Complexes with IL-6R to inhibit infection	Zuo et al. (33)
SeV	Primary human keratinocytes	Activation of STAT1 leads to *OAS2* transcription independent of type I and type II IFNs	Kwock et al. (19)
	Mice	Mediates immunopathology by promoting T-cell production of IL-10	Muallem et al. (41)
DENV	Primary human cell co-cultures	Production by DCs promotes T_FH_ cell differentiation, supporting B-cell antibody production	Sprokholt et al. (42)

## Interferon-Dependent Mechanisms

Interferons (IFNs) are a class of antiviral cytokines produced by a variety of cell types, such as macrophages and dendritic cells (DCs), in response to PRR stimulation. Three families of IFNs have been characterized: type I IFNs (IFNα/β), type II IFNs (IFNγ) and type III IFNs (IFNλ1, 2, 3, and 4). Signaling by type I and III IFNs induces STAT1 and STAT2 phosphorylation and dimerization, with the resulting STAT1/STAT2 complex interacting with IFN regulatory factor-9 (IRF9) to form ISG factor 3 (ISGF3) (45). ISGF3 translocates to the nucleus and binds to IFN-stimulated response elements (ISRE) to activate transcription of antiviral genes known as interferon stimulated genes (ISGs). Similarly, type II IFNs induce several ISGs through homodimerization of phosphorylated STAT1 which binds gamma activated sequence (GAS) elements in the nucleus (45). ISGs encompass a broad range of genes whose products inhibit some stage of the viral life cycle. For instance, bone marrow stromal cell antigen 2 (BST-2) inhibits budding of several enveloped viruses (46), whereas myxovirus resistance protein 1 (MX1) inhibits viral transcription (47). A detailed review of ISG production and function is beyond the scope of this paper and can be reviewed in greater detail here (45, 48, 49).

**Figure 1:**
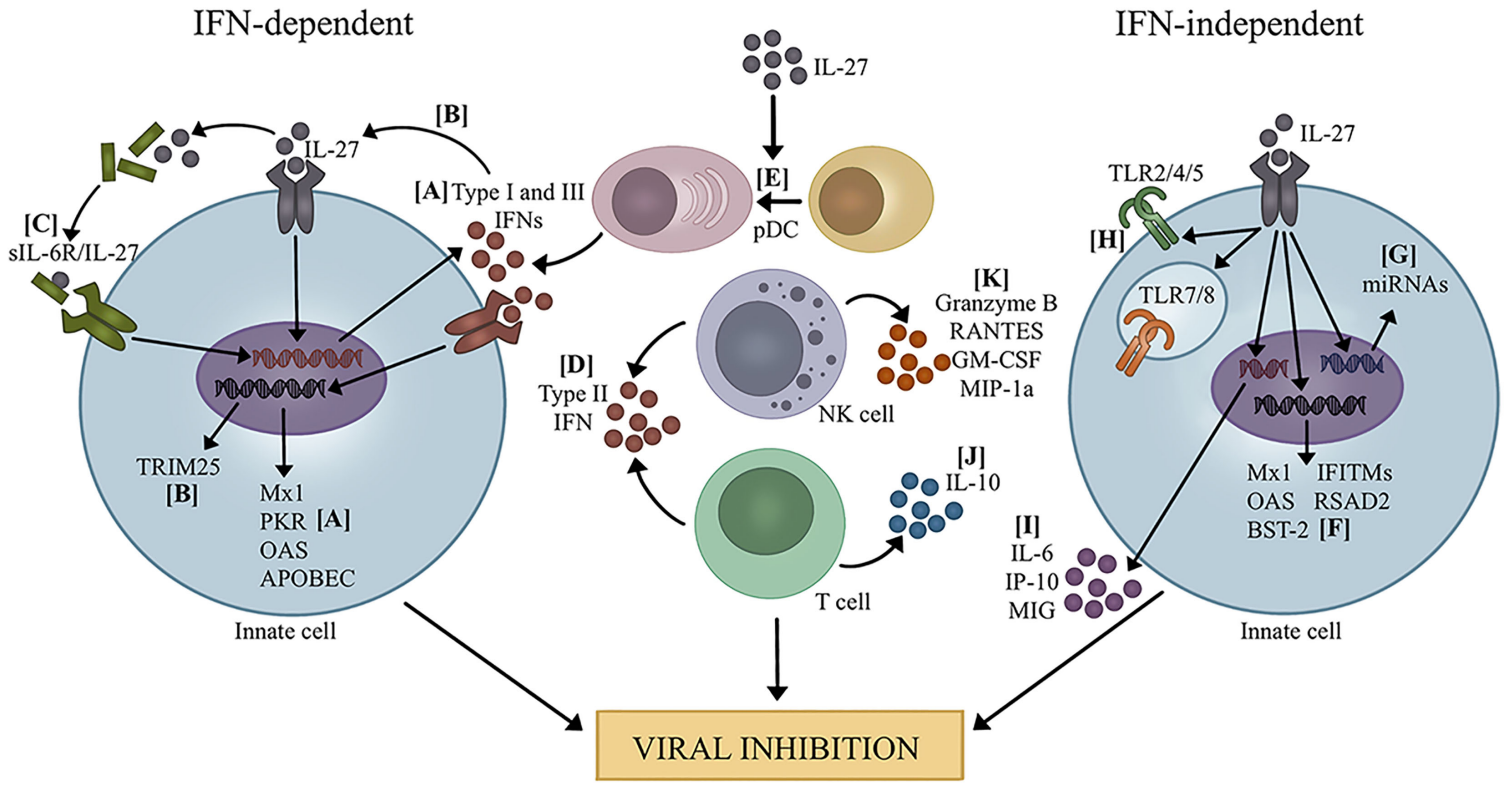
Mechanisms of viral inhibition by IL-27. IFN-dependent: **(A)** IL-27 promotes the production of type I and III IFNs which leads to transcription of ISGs (20, 23, 29). **(B)** IL-27 induced by type I IFNs leads to expression of TRIM25 (21). **(C)** IL-27 complexes with sIL-6R and increases production of type I and III IFNs, leading to transcription of several ISGs (31, 33, 34). **(D)** IL-27 promotes the production of type II IFN by T cells and NK cells (28, 35, 51–54). **(E)** IL-27 promotes pDC differentiation, leading to increased IFN production (39). IFN-independent: **(F)** IL-27 directly promotes the transcription of several ISGs (19, 25, 27, 32, 38, 55). **(G)** IL-27 induces the expression of miRNAs that potentially target several viruses (56, 57). **(H)** IL-27 enhances the expression and signaling capacity of TLRs, which could influence recognition of PAMPs and DAMPs during viral infection (10–15). **(I)** IL-27 increased pro-inflammatory cytokine and chemokine production (40). **(J)** IL-27 promotes IL-10 production by T cells to mediate immunopathology during viral infection (18, 36, 37, 41, 58–61). **(K)** IL-27 enhances NK cell function by increasing granzyme B, RANTES, GM-CSF, and MIP-1α (28, 39).

Activation and production of IFNs and IL-27 appear connected. Clinical studies demonstrate that IL-27 and IFN levels are tightly correlated during viral infection (20, 50). Additionally, *in vitro* and *in vivo* studies confirm that IL-27 directly induces IFN production and vice versa by various cell types, such as DCs, macrophages, NK cells, hepatocytes, and lung epithelial cells (18, 20, 28, 29, 36, 39, 40, 43, 44). As such, much of the antiviral activity associated with IL-27 has been attributed to its augmentation of IFN production (**Figure 1**).

### IL-27 Induction of IFNs Enhances ISG Transcription

Accumulating evidence supports the proposal that IL-27 enhances ISG transcription during viral infection by augmenting the production of IFNs. For instance, IL-27 enhances IFNα and IFNλ1 production by human hepatocytes during HBV infection, leading to increased expression of ISGs, such as 2’-5’-oligoadenylate synthetase 1 (*OAS1*), *MX1* and protein kinase R (*PKR*), and inhibition of HBV replication (20). Promotion of extracellular signal-regulated kinase 1 (ERK1)/ERK2 signaling as well as nuclear factor kappa B (NFκB) nuclear translocation by IL-27 was found to regulate IFN- λ1 expression (20). Inhibition of these IFNs using an RNA interference approach ablated this effect, suggesting that the antiviral effects of IL-27 were mediated by the enhanced production of IFNα and IFNλ1 (20). The addition of type I IFN neutralizing antibodies similarly reduced the ability of IL-27 to inhibit influenza A virus (IAV) infection in human peripheral blood mononuclear cells (PBMCs) (29). Neutralizing IFNα/β reduced phosphorylation of STAT1 and STAT2, along with reduced phosphorylation of the ISG PKR, demonstrating that the type I IFNs induced by IL-27 activated PKR to limit IAV infection in this model (29). One study compared the effects of adding IL-27 or IFNα to HIV-infected primary human monocyte-derived macrophages (MDMs) (23). Addition of IL-27 or IFNα separately inhibited HIV through induction of host restriction factors apolipoprotein B mRNA editing enzyme catalytic polypeptide (APOBEC) cytidine deaminases. However, the kinetics of this induction differed, with a delayed synthesis of APOBEC cytidine deaminases observed with IL-27 compared to IFNα. Interestingly, IL-27 was found to induce IFNα and use of IFNα/β-receptor neutralizing antibodies revealed that intermediate IFNα was required to inhibit HIV replication (23).

*In vitro* and clinical studies show that IL-27 can interact with IL-6 to form a complex during viral infection (33). IL-6 is a pleiotropic cytokine produced in response to infection and tissue damage that modulates numerous antiviral responses such as T cell and macrophage activity [reviewed here (62)]. The IL-27p28 subunit of IL-27 has been demonstrated to form a complex with soluble IL-6 receptor (sIL-6R) and induce STAT1 and STAT3 signaling in IL-27 responsive cells (63). This IL-6/IL-27 complex exerts antiviral effects against a number of viruses, including influenza, Sendai virus (SeV), vesicular stomatitis virus (VSV), HSV-1, enterovirus 71 (EV71), and HBV (31, 33, 34). Mechanistically, the IL-6/IL-27 complex inhibited viral infection by promoting type I and III IFN production which led to downstream activation of ISGs such as *OAS1*, *PKR*, and *MX1 (* 31, 33, 34). Induction of IFNs was due to IL-6/IL-27 complex interaction with the mitochondrial antiviral signaling protein (MAVS)/TNF receptor-associated factor 3 (TRAF3)/TRAF6 pathway, leading to subsequent nuclear translocation of NFκB (31, 33). Of the two IL-27 subunits, IL-27p28 was found to mediate much of the antiviral response, as significant reductions in viral inhibition and IFN and ISG expression were reported when IL-27p28 was silenced using RNA interference (33, 34). Similar reductions in IFN and ISG expression were observed when EBI3 was silenced, however, this decrease was not as robust as in the absence of IL-27p28 (33). Nonetheless, increased viral inhibition was observed upon treatment with the IL-6/IL-27 complex compared to the individual subunits of the complex (31, 33), suggesting that the sIL-6R subunit contributes to the antiviral effects as well. Moreover, the IL-27p28 subunit (also known as IL-30) can act independently from EBI3 (6) and IL-27p28 bound to sIL-6R without EBI3 does exert antiviral effects (31). However, addition of an IL-27p28 expressing plasmid had no effect on the viral replication of IAV, EV71, or HBV (31), suggesting that the interaction of IL-27p28 with sIL-6R and/or EBI3 promotes the antiviral activities of IL-27p28. Further investigation into the mechanism behind the antiviral functions of the IL-6/IL-27 complex is therefore warranted.

Increased ISG transcription as a consequence of IL-27-induced IFNs can contribute to effective viral clearance. However, this antiviral defense does not counteract the numerous anti-IFN mechanisms that viruses have developed to interfere with ISG production, such as inhibiting IFN signaling (64). This strategy may therefore be most beneficial to uninfected bystander cells where IFNs can signal unimpeded by viral inhibitory mechanisms to promote antiviral states. Alternatively, IL-27 induction of IFNs by infected cells could be enough to overcome viral inhibition of IFNs and could then result in robust ISG transcription.

### IL-27 Promotes IFNγ Production

IFNγ is a pleiotropic cytokine produced predominantly by activated NK cells and T cells. Beyond promoting ISG transcription, IFNγ-mediated augmentation of innate immune responses, including antigen presentation, makes it a key link between innate and adaptive responses during infection (65). A growing body of evidence demonstrates that IL-27 signaling promotes IFNγ production by CD8+ T cells during viral infection. *In vitro* data demonstrates that STAT1 and T-bet activation induced by IL-27 treatment concurrently with IL-12 augments IFNγ production by CD8+ T cells (51–53). CD8+ T cell effector functions, such as granzyme B and perforin production, are also increased by IL-27 (51, 52). Consistent with these data, IL-27 receptor (IL-27R) and T-bet signaling were found to be critical for IFNγ production *in vivo* during viral infection (35, 54). A lack of IL-27 signaling resulted in reduced expression of IFNγ and T-bet in the lungs of respiratory syncytial virus (RSV) infected *il27rα-/-* mice compared to infected WT controls (54). In mixed bone marrow (BM) chimera mice generated from *il-27rα-/-* and WT donors, a reduced frequency of IFNγ producing IAV antigen-specific *il-27rα-/-* CD8+ T cells compared to internal WT controls were isolated from spleens and lymph nodes following IAV infection (35). The production of IFNγ-producing, HCV-specific CD8+ T cells in the spleens of mice was similarly enhanced by coadministration of either an IL-23 or IL-27 and an IL-12 expression plasmid following immunization (17). The promotion of IFNγ-producing antigen-specific CD8+ T cells by IL-27 highlights the adjuvant potential of this cytokine for prophylactic measures against viral infection.

*In vitro* studies with primary human NK cells demonstrated that IL-27 can work synergistically with IL-15 and/or IL-18 to promote IFNγ secretion by NK cells (66, 67). In a mechanism similar to CD8+ T cells, IL-27 induction of STAT1 and T-bet activity have been shown to be involved in this process (67). In the context of viral infection, NK cells from *ebi3-/-* and *il27Ra-/-* mice exhibited significant reductions in IFNγ production during the early phase of IAV infection compared to WT controls (28). In this study, stimulation of NK cells *in vitro* with IL-27 alone had no effect on IFNγ production. However, IL-27 significantly augmented NKG2D activation of NK cells and subsequent IFNγ production, suggesting that IL-27 may act as an additional signal alongside IL-15 or IL-18 to enhance NK cell activation at the site of viral infection (28).

A clinical study aligns with these data, as a positive correlation between IL-27 and IFNγ plasma levels in CMV-infected patients has been observed (50). However, contradictory findings of whether IL-27 promotes IFNγ production by T cells and NK cells have been reported. Increased IFNγ-producing T cells and NK cells in virally infected *il27ra-/-* mice have been observed compared to their WT counterparts (18, 39). The timing of IL-27 activity may be critical as to whether it increases or decreases IFNγ production systemically during viral infection. Early IL-27 signaling may promote IFNγ production to increase viral clearance. However, IL-27 also promotes IL-10 production, (reviewed in a later section) which could downregulate IFNγ production later during infection to mediate immunopathology.

### IL-27 Supports pDC Differentiation

Plasmacytoid DCs (pDCs) are a subset of DCs that specialize in sensing viral DNA and RNA, upon which IFNs are rapidly produced (68). Following production of IFNs, pDCs help shape the adaptive immune response by stimulating T cells (68). IL-27 was shown to support the development of pDCs in mice during viral infection (39). In *il27ra-/-* mice, a lack of pDC expansion was observed during chronic LCMV infection, and this was associated with reduced type I IFN levels and increased viremia compared to WT controls (39). As a result, LCMV infected *il27ra-/-* mice suffer from higher viral load compared to their WT counterparts. Moreover, a significant reduction in CD86, a key costimulatory molecule for T cell activation, was found in pDCs and other DC subsets from chronically LCMV infected *il27ra-/-* mice (39). Together, these data suggest that IL-27 promotes pDC differentiation and in the absence of IL-27 signaling, pDC numbers and function are reduced, which potentially leads to inefficient IFN production and T cell activation. The factors that regulate pDC specification are complex and the way in which IL-27 promotes this differentiation is unknown. However, there is evidence that IL-27 can promote the expression of the transcription factor interferon regulatory factor 8 (IRF8) (69), which has been suggested as one of the initiators of pDC differentiation (68).

### Type I IFNs Promote IL-27

The axis between IL-27 and type I IFNs is bidirectional, with type I IFN-induced IL-27 also promoting antiviral activities. Type I IFNs directly augment IL-27 production *via* IRF-1 initiation of IL-27p28 subunit transcription (44, 70). During HBV infection, type I IFN-mediated gene and protein production of the ISG tripartite motif containing 25 (TRIM25) was found to be dependent upon intermediate IL-27 signaling (21). IFNα conditioned DCs were highly efficient at cross-priming CD8+ T cells with viral antigen, and this was associated with an increased potential to express IL-23 and IL-27 (43). IL-27 enhances HLA-ABC and HLA-DR expression in DCs (25), therefore, IFNα-induced IL-27 may result in augmented antigen presentation in DCs. IL-27 produced as a consequence of type I IFN signaling has also been shown to influence T cell responses during viral infection (36, 37, 42, 58). For instance, type I IFN-induced IL-27 production by primary human DCs in response to dengue virus (DENV) infection was critical for T follicular helper (T_FH_) cell differentiation, consequently supporting secretion of antibodies by activated B cells (42). Further discussion of T cell function influenced by type I IFN-mediated IL-27 will be done below (see section 4.3).

## Interferon-Independent Mechanisms

Studies have shown that inhibition of IFNs using neutralizing antibodies does not fully ablate viral inhibition upon IL-27 treatment (20, 26, 29), which suggests that IL-27 has other IFN-independent mechanisms of viral control. For example, mice knock-out (KO) experiments support this idea, as subcutaneous infection with ZIKV causes 50% mortality in *ifnar1-/-* mice but 100% mortality in *ifnar1-/-il27ra-/-* mice, demonstrating additional antiviral activities of IL-27 beyond IFN modulation (19).

### Direct Induction of ISGs by IL-27

Induction of ISG transcription is not exclusive to IFNs and can be done by any substrate that activates interferon regulatory factors (IRFs) (45). Accumulating evidence suggests that along with inducing IFNs, which signal to promote IGS transcription, IL-27 can also directly induce ISG production (**Figure 1**). For instance, IL-27 inhibited HIV infection in primary human monocyte-derived DCs and macrophages through induction of several ISGs including *MX1*, *OAS2*, *OAS3*, interferon-induced transmembrane protein 1(*IFITM1*), *IFITM3*, radical S-adenosyl methionine domain containing 2 (*RSAD2*), and *PKR*, even in the presence of type I IFN neutralizing antibodies (25, 32). BST-2, a key inhibitor of HIV infection, was upregulated in primary human monocytes and T cells at both the gene and protein level in response to IL-27 treatment (55, 71). Addition of the vaccinia virus-encoded type I IFN decoy receptor B18R abrogated IFN-mediated induction of BST-2, but had no effect on IL-27-mediated BST-2 expression, demonstrating that BST-2 induction by IL-27 is independent of intermediate type I IFN production (55). Knockdown of IFN receptors IFNAR1 and IFNGR1 in primary human keratinocytes similarly had no effect on IL-27-induced upregulation of *OAS2* expression during ZIKV and Sendai virus infection (19). Moreover, HepG2 cells infected with avian and human influenza upregulate *MX1* to a greater extent following IL-27 treatment, and this was unaffected by the presence of IFNα and IFNγ neutralizing antibodies (27). In a model of chikungunya virus (CHIKV) infection of primary human MDMs, there is a lack of expression of all three IFN types, despite a robust antiviral response being observed (38). Expression of IL-27 on the other hand, is significantly increased, and IL-27 treatment dose-dependently inhibited CHIKV replication. Investigation of THP-1 derived macrophages revealed that IL-27 promoted ISG production, such as *IFITMs*, *MX1*, *MX2*, *PKR*, and *RSAD2*. Therefore, IL-27 might be responsible for the anti-CHIKV response in the absence of IFNs (38). The direct induction of ISGs by IL-27 may be a mechanism to prevail over virus-mediated inhibition of IFN signaling and ISG transcription. In other words, IL-27 may serve as a backup antiviral response in the absence of efficient IFN function.

Similar to IFNs, IL-27 relies on JAK-STAT signaling for ISG production. Phosphorylation of STATs 1, 2, and 3 is frequently reported in the context of viral infection (19, 20, 25, 27, 29, 72). STAT1/STAT3 heterodimerization is favoured by IL-27 during viral infections such as HBV and HSV (21, 40, 73), and may represent an alternative pathway to ISG production, as the STAT1/STAT3 heterodimer is not typically associated with IFN signaling. There is limited evidence that IL-27 signals through STAT1/STAT2, thus increased production of this heterodimer is likely due to IL-27-induced type I and III IFN production. Indeed, in the absence of type I IFNs, IL-27-mediated STAT1/STAT2 phosphorylation was significantly reduced, suggesting that enhanced phosphorylation of this heterodimer was due to IL-27-induced type I IFN production (29). Significant STAT1 phosphorylation and STAT1/STAT1 homodimerization during viral infection has also been reported (19, 27). There is evidence that, IL-27 signals *via* STAT1/STAT1 dimers (74) and therefore may do so to directly induce ISG transcription. Notably, type II IFNs also signaling through STAT1 homodimers, thus the potential of type II IFNs being involved in IL-27-induced STAT1 homodimerization cannot be completely ruled out. The redundancy in signaling by IL-27 and type II IFNs helps explain the overlapping profiles of ISGs induced by IL-27 and IFNs (32, 53, 75). However, the similarity in IFN and IL-27 signaling also raises the question as to whether viral mechanisms developed to interfere with IFN signaling may affect IL-27 signaling as well. Clinical studies support this notion, as a negative correlation between IL-27 serum and plasma expression and viral load was reported in HIV-positive and HIV-HCV co-infected patients (76, 77). *In vitro*, HIV infection and the HIV protein Tat inhibited LPS-induced IL-27 production in primary human MDMs (78). These studies suggest that HIV inhibits IL-27 as a mechanism of immune suppression. However, contradictory findings from clinical studies have been observed, with one study reporting a positive correlation (79) and another reporting no relationship between IL-27 and HIV viral load (80). Differences in severity of infection regardless of viral load could account for these contradictions, however, further investigation into whether HIV impedes IL-27 production is needed. Nonetheless, HIV could interfere with IL-27 function rather than production. For instance, PBMCs isolated from HIV patients had impaired responses to IL-27, with downregulation of IL-27-induced IL-6, TNFα, and IL-10 compared to PBMCs from healthy controls (81). Whether other viruses have the capacity to interfere with IL-27 production and signaling remains to be determined.

### IL-27 Modulates the Antiviral Innate Response

Beyond IFN and ISG induction, IL-27 has been shown to promote antiviral responses by influencing the effector functions of innate immune cells (**Figure 1**). For instance, primary human monocytes differentiated into macrophages in the presence of IL-27 (referred to as I-Macs) produced several microRNA (miRNAs) demonstrated to target the open-reading frames of viruses such as HSV-1, HSV-2, and HHV-8 (56). Similar miRNAs with antiviral potential were observed in primary human DCs in response to IL-27 treatment (57). I-Macs also displayed an HIV-resistant phenotype characterized by reduced levels of the HIV-supportive host factor spectrin B nonerythrocyte 1 (SPTBN1) (24). Blocking IFNα with neutralizing antibodies had no effect on I-Macs resistance to HIV, suggesting that IL-27 rather than intermediate IFNα production was responsible for the HIV-resistant phenotype. Resistance to other viruses, such as influenza, SIV, and KSHV, was also observed by I-Macs compared to primary human monocytes differentiated into macrophages without IL-27 (24). Moreover, IL-27 treatment influences cytokine production. Following IL-27 treatment, enhanced secretion of cytokines IL-6, IP-10 (CXCL10), and MIG by macrophages, DCs, and human epithelial cell lines was observed along with restricted HSV-1 replication (40). Augmentation of proinflammatory cytokine production by IL-27 may help promote inflammation and immune cell recruitment needed for viral clearance. However, a balance in proinflammatory cytokine production is needed in order to mitigate the risk of unregulated inflammation and detrimental cytokine storm.

Going hand in hand with macrophage differentiation, IL-27 may also influence macrophage polarization, which could have implications for viral inhibition. Briefly, environmental stimuli induce different polarization states in macrophages. In general, M1 macrophages display pro-inflammatory and antiviral properties and have been shown to be more resistant to viral infection, whereas M2 macrophages display anti-inflammatory properties and are more permissive to infection but important for mediating immunopathology (82, 83). There is evidence to suggest that IL-27 skews macrophage polarization to the M1 phenotype (69, 84, 85); therefore, IL-27 may reduce the ability of viruses to infect macrophages by promoting M1 polarization. Genetic analysis of HIV-resistant I-Macs (monocytes treated with IL-27 during differentiation to macrophages) revealed an upregulation of M1 markers CD80 and TNF (24). Further, primary human M1-like macrophages were found to be potent producers of type I and III IFNs during rhinovirus infection *in vitro (* 86), therefore, by promoting M1 polarization, IL-27 may increase IFN production as well. However, a direct connection between IL-27, macrophage polarization, IFN production, and viral infection has not yet been studied in detail.

Recent insights demonstrate that IL-27 can modulate the function of TLRs, an important class of PRRs involved in recognizing viral components. Augmented signaling capacity and resulting cytokine production by cell surface TLR2, 4, and 5 on human monocytes and macrophages (primary and cell lines) has been observed as a result of IL-27 treatment (10–14). Intracellular TLR7 and 8 are similarly affected by IL-27, with enhanced TLR7 expression and TLR8-mediated cytokine production observed upon treatment with IL-27 in THP-1 monocytes and THP-1-derived macrophages (15). Recognition of viral PAMPs, such as viral DNA and RNA, and damage-associated molecular patterns (DAMPs) from dead or dying cells by these TLRs is essential for activating innate immunity against a variety of viruses (87). Therefore, IL-27-mediated increase of TLR expression and signaling may enhance recognition of viruses and induce a more robust antiviral response. Moreover, several viruses, such as HCV, human cytomegalovirus (HCMV), HSV, Kaposi’s sarcoma-associated herpes virus (KSHV), and vaccinia virus have evolved mechanisms of inhibiting TLR function (88–96). The increase in TLR expression and function by IL-27 could help counteract these inhibitory mechanisms. Currently, the potential relationship between IL-27 modulation of TLRs and viral immune responses has not been elucidated.

Another way that IL-27 promotes innate immune responses during viral infection is by augmenting NK cell function. Beyond promoting IFNγ production, IL-27 also enhances the production of GM-CSF, RANTES, and MIP-1α by NK cells following NKG2D-mediated activation (28). The chemoattractant properties of RANTES and MIP-1α promote the migration of immune cells to the site of infection, including memory and effector T cells, granulocytes, and macrophages (97, 98). GM-CSF is a growth factor responsible for the survival, proliferation and activation of immune cells such as macrophages and DCs (99). Increased infiltration and activation of immune cells at the site of infection in response to these cytokine and chemokines could support viral clearance. However, enhanced immune cell infiltration has been linked to cytokine storm and worse outcomes during infections such as IAV (100), therefore, a balance is needed to ensure effective viral clearance without causing severe immunopathology. Additionally, NK cell production of granzyme B, a protease that kills virus-infected cells, was diminished in *il27rα-/-* mice and associated with reduced viral clearance, suggesting a role of IL-27 in mediating NK cell effector functions during virus infection (28, 39).

### IL-27 Promotes IL-10 Production to Mediate Immunopathology During Virus Infection

Much of the morbidity and mortality caused by viral infections can be attributed to overactive immune responses including overproduction of cytokines, infiltration of immune cells and excessive inflammation that damages the body. As such, balancing these strong effector responses is critical to limiting immunopathology. IL-10 is an important immunoregulatory cytokine that mediates this balance in the immune response, as it can suppress both innate and adaptive immune responses to viral infections (101). However, certain viruses can also exploit the immunomodulatory functions of IL-10 to establish chronic infection (102–104). While various innate and adaptive immune cell types have been identified as IL-10 producers, CD4+ and CD8+ T cells are important sources of IL-10 during viral infections (105, 106).

There is increasing evidence that IL-27 is a potent inducer of IL-10 production from CD4+ and CD8+ T cells in a variety of viral infections, thereby having implications on antiviral immune responses and viral clearance (**Figure 1**). Significant reductions in IL-10-producing CD4+ and CD8+ T cells are observed during viral infection in *il27ra-/-* and *ebi3-/-* mice compared to their WT counterparts (18, 37, 41, 58, 59). Indeed, a study by Perona-Wright and colleagues (60) demonstrated that direct IL-27 signaling was critical for IL-10 production by CD8+ T cells. When mice with mixed bone marrow from WT and IL27Ra-/- congenic bone marrow were infected with Sendai virus, WT but not the IL-27R-deficient CD8+ T cells produced IL-10 (60). Mechanistically, influenza infection models have demonstrated that IL-27 augments IL-10 production by CD8+ T cells synergistically with IL-2 by promoting and sustaining expression of IRF4 and B-lymphocyte maturation protein-1 (Blimp-1) (36, 37). On the other hand, IL-27 activation of STAT4 was found to partially mediate IL-10 production by murine CD4+ T cells (18). The increases in IL-10 by IL-27 appears to be partially reliant on type I IFNs. Type I IFN signaling was reported to enhance IL-27 secretion by myeloid cell populations during MCMV and IAV viral infections, which in turn promoted IL-10 production by T cells *in vivo* (36, 37, 58). *In vitro* data demonstrate a more direct role of type I IFNs, as the addition of IFNα greatly enhanced IL-10 secretion by CD8+ T cells in conjunction with IL-27 and IL-2 treatment (36). Thus, the increase in IL-10 expression by IL-27 may not be an entirely IFN-independent mechanism. However, other cytokines can also promote IL-27-mediated IL-10. For instance, IL-6 has been reported to be involved in inducing IL-10, as IL-6 enhanced IL-27 production by murine macrophages and monocytes during RSV infection which in turn promoted T-regulatory cell (Treg)-derived IL-10 (61).

There are mixed reports as to whether IL-27-induced IL-10 increases or decreases survival following viral infection. Increased IL-10-producing T cells due to IL-27 was found to enhance survival during infection with viruses such as IAV, RSV, and SeV by attenuating immune cell infiltration, cytokine production, and inflammation (18, 41, 61). However, IL-27-induced IL-10-producing T cells have also been reported to promote viral persistence during murine cytomegalovirus (MCMV), mouse hepatitis virus (MHV) strain murine hepatitis virus (JHMV), and IAV infection by over suppression of the immune responses leading to ineffective viral clearance (18, 58, 59). The aspect that appears critical to whether the IL-27-induced IL-10 response attenuates or augments viral infection is timing. Early IL-27 signaling induces IL-10 which downregulates the immune response too quickly, leading to viral spread (18, 58, 59). However, if IL-27 induces IL-10 at a later time during infection, immunopathology is reduced while preserving the antiviral immune response (18, 41, 61). The timing of IL-27 function during virus infection appears paradoxical, as early IL-27 signaling is important for enhancing innate antiviral responses and potentially regulating IFNγ production, but also may attenuate these very responses by increasing IL-10. The availability of IL-27, the cell types responding and the kinetics of virus infection all may come into play to determine whether IL-27 signaling enhances or attenuates antiviral immune responses.

## Discussion

The importance of IFNs in establishing a successful antiviral response is indisputable. However, strong selective pressure from IFNs has led to the evolution of a variety of viral IFN-inhibitory mechanisms that allow viruses to effectively establish infection (64, 107). Alternative pathways have developed in order to keep up with this virus-immune response evolutionary arms race. IL-27 may represent one of these alternative pathways. As an inducer of type I, type II, and type III IFNs (17, 20, 23, 28, 29, 35, 51, 52, 54), IL-27 may augment IFN production such that a robust antiviral response still occurs despite viral IFN-inhibitory mechanisms. It is interesting to speculate whether IL-27 has a dominant role in the induction IFNs in general during viral infection. There is evidence for mutual direct induction between IL-27 by IFNs (20, 44, 70). However, as numerous stimulants promote IFN and IL-27 production during viral infection, such as viral PAMPs, DAMPs, and other cytokines, the role of IL-27 in promoting IFN production may be supportive, rather than dominant. Further, IL-27 has overlapping functions with IFNs, and can directly promote ISG transcription (19, 25, 27, 32, 55, 71). Being independent of IFNs, IL-27 signaling may be able to bypass the IFN-inhibitory mechanisms of viruses and successfully induce ISG transcription. However, IL-27 signaling pathways are similar to that of IFNs, making it plausible that viral mechanisms developed to interfere with IFN signaling may be effective at hindering IL-27 signaling as well.

Overall, the interwoven relationship between IFNs and IL-27 makes it difficult to delineate their antiviral mechanisms as entirely separate. The most compelling evidence that IL-27 also acts independently of IFNs is that in IFN-deficient models, inhibition of viral replication still occurs upon IL-27 treatment (25, 26, 29). Indeed, these IFN-independent mechanisms include acting as a IFN itself and directly inducing ISGs, but also modulating other immune responses such as proinflammatory cytokine and chemokine production (28, 40), macrophage and DC differentiation (24, 39), and IL-10 anti-inflammatory function (18, 36, 37, 41, 58–61). The vast array of responses influenced by IL-27 relates to its ability to inhibit a variety of viral infections and makes it a promising therapeutic and vaccine adjuvant for existing and emerging viral threats.

## Author Contributions

HA and KG conceptualized this review; HA led the writing of this review with OK and MR contributing equally to information on the biology of IL-27 and on IL-27 and T cell responses respectively. HA designed the figure and table. OK was responsible for the initial editing of the review and HA was responsible for combining edits from all authors. KG oversaw all aspects or writing editing and submitting the review. All authors contributed to the article and approved the submitted version.

## Funding

This work was supported by funding from the Natural Sciences and Engineering Research Council of Canada (NSERC), grant number: RGPIN-2017-04526.

## Conflict of Interest

The authors declare that the research was conducted in the absence of any commercial or financial relationships that could be construed as a potential conflict of interest.

## Publisher’s Note

All claims expressed in this article are solely those of the authors and do not necessarily represent those of their affiliated organizations, or those of the publisher, the editors and the reviewers. Any product that may be evaluated in this article, or claim that may be made by its manufacturer, is not guaranteed or endorsed by the publisher.
